# Elemental Composition of Magnetic Nanoparticles in Wildland–Urban Interface Fire Ashes Revealed by Single Particle-Inductively Coupled Plasma-Time-of-Flight-Mass Spectrometer

**DOI:** 10.3390/nano15181420

**Published:** 2025-09-15

**Authors:** Mahbub Alam, Austin R. J. Downey, Bo Cai, Mohammed Baalousha

**Affiliations:** 1 Center for Environmental Nanoscience and Risk, Department of Environmental Health Sciences, Arnold School of Public Health, University of South Carolina, Columbia, SC 29208, USA; mahbub@email.sc.edu; 2 Department of Mechanical Engineering, University of South Carolina, Columbia, SC 29208, USA; 3 Department of Civil and Environmental Engineering, University of South Carolina, Columbia, SC 29208, USA; 4 Department of Epidemiology and Biostatistics, Arnold School of Public Health, University of South Carolina, Columbia, SC 29208, USA; bocai@mailbox.sc.edu

**Keywords:** wildland–urban interface, fires, magnetic nanoparticles, elemental composition, SP-ICP-TOF-MS, NMR

## Abstract

This study investigates the elemental composition of magnetic nanoparticles (MNPs) in eleven wildland–urban interface (WUI) fire ashes, including one vegetation, six structural, and four vehicle ashes, along with three fire-impacted soil samples. The WUI fire ash samples were collected following the 2020 North Complex (NC) Fire and Sonoma–Lake–Napa unit (LNU) Lightning Complex Fire in California. Efficiency of magnetic separation was confirmed via Time-Domain Nuclear Magnetic Resonance (TD-NMR); the relaxometry showed that the transverse relaxation rate R_2_ decreased from 2.02 s^−1^ before separation to 0.29 s^−1^ after separation (ΔR_2_ = −1.73 s^−1^; −86%), due to the removal of magnetic particles. The particle number concentrations, size distributions, and elemental compositions (and ratios) of MNPs were determined using single particle-inductively coupled plasma–time-of-flight-mass spectrometry (SP-ICP-TOF-MS). The major types of nanoparticles (NPs) detected in the magnetically separated MNPs were Fe-, Ti-, Cr-, Pb-, Mn-, and Zn-bearing NPs. The iron-bearing NPs accounted for 3.2 to 83.5% of the magnetically separated MNPs, and decreased following the order vegetation ash (77.4%) > soil (63.2–69.9%) > structural (3.2–83.5%) ash. The titanium-bearing NPs accounted for 3.3 to 66.1% of the magnetically separated MNPs, and decreased following the order vehicle (14.1–66.1%) > structural (3.5–36.4%) > vegetation (3.3%) ash. The majority of the detected NPs in the fire ashes occurred in the form of multi-metal (mm) NPs, attributed to the presence of NPs as heteroaggregates and/or due to the sorption of metals on the surfaces of NPs during combustion. However, a notable fraction (3–91%) of the detected NPs occurred as single-metal (sm) NPs, particularly smFe-bearing NPs, which accounted for 48 to 91% of all the Fe-bearing particles in the magnetically separated MNPs. The elemental ratios (e.g., Al/Fe, Ti/Fe, Cr/Fe, and Zn/Fe) in the magnetically separated MNPs from structural and vehicle ashes were higher than those in the soil samples and vegetation ashes, indicating enrichment of metals in magnetically separated NPs from vehicle and structural ashes compared to vegetation ash. Overall, this study demonstrates that the MNPs generated by WUI fire ash are associated with potentially toxic elements (e.g., Cr and Zn), exacerbating the environmental and human health risks of WUI fires. This study also highlights the need for further research into the properties, environmental fate, transport, and interactions of MNPs with biological systems during and following WUI fires.

## 1. Introduction

Wildland–urban interface (WUI) fires, which occur at the boundary zones between wilderness areas and human habitation, represent a significant environmental and societal concern due to their destructive nature [1] and increasing frequency fueled by climate change [2,3]. These fires not only pose immediate threats to lives and property, but also leave behind a complex residue of ashes that can have long-lasting ecological and health implications [3,4]. WUI fires alter various fuels, such as vegetation, soil organic matter, and construction materials, leading to the formation of substances with altered chemical and physical properties. These include black carbon, methane, carbon monoxide, carbon dioxide, and ash [4]. Fire emissions, including black carbon, methane, and carbon monoxide, along with the intense heat generated, create a highly reducing environment in the surrounding areas [5,6]. The highly reducing environment of WUI fires leads to the conversion of metal oxides into suboxides. For instance, previous studies have demonstrated the presence of reduced iron oxides (i.e., magnetite, wüstite, and zero-valent iron) in WUI fire ash [7]. Other studies have demonstrated the reduction of TiO_2_ to titanium suboxides, such as the Magnéli phases (Ti_n_O_2n−1_, 4 < *n* < 9), in coal-fired power plants [8,9].

Several studies have highlighted the release of diverse classes of contaminants due to WUI fires, including environmentally persistent free radicals, metals, metalloids, magnetic particles, nutrients, and other substances [7,10,11,12,13,14,15,16,17]. Among the diverse array of contaminants of concern in WUI fire ash are magnetic nanoparticles (MNPs), due to their unique properties and potential environmental and human health impacts [7,18,19,20]. Several studies have demonstrated that the combustion of vegetation and structural materials and the heating of soil during fires leads to the formation of magnetite particles [7,21,22,23]. However, comprehensive data regarding the concentrations and elemental makeup of MNPs formed in WUI fires remain limited [7].

Magnetic particles found in fire ash, particularly iron-containing particles (i.e., magnetite), pose significant risks to human health and the environment. Various studies have detected airborne magnetite nanoparticles in different human organs, suggesting the potential for atmospheric magnetite to enter the human circulatory system or even penetrate brain tissue [18,24,25]. Exposing human lung cells to different sizes and doses of magnetite particles, including nanoparticles, led to the increased formation of reactive oxygen species, mitochondrial damage, and genotoxic effects [26]. The presence of magnetite nanoparticles in the brain has been potentially linked to several neurodegenerative diseases, such as Alzheimer’s and Parkinson’s diseases, as oxidative stress plays a critical role in their development [19,20]. Additionally, magnetite nanoparticles may have various environmental impacts, such as affecting soil magnetism [22,23], fostering the formation of algal blooms [27,28], and contributing to climate change through the absorption of solar radiation [29,30].

An in-depth characterization of MNPs in WUI fire ash is crucial for unraveling the intricate processes involved in their formation, transport, environmental fate, and environmental and human health effects. To this end, we investigated the magnetic separation and characterized the properties (e.g., number concentrations, size distributions, elemental compositions, and elemental ratios) of MNPs in WUI fire ashes using single particle-inductively coupled plasma–time-of-flight-mass spectrometry (SP-ICP-TOF-MS). Through a combination of high-resolution elemental analysis and statistical tools, such as cluster analysis, we provide a detailed assessment of MNPs in WUI fire ashes. Therefore, our findings provide valuable insights into the nature and properties of MNPs in WUI fire ashes, enhancing our understanding of their pollution characteristics and environmental health risks.

## 2. Materials and Methods

### 2.1. Sampling Location and Sample Collection

Eleven WUI fire ash samples, including structural (A12, A24, A92, A124, A135, and NC-2), vehicle (A13, NC-4C, NC-11A, and NC-11B), and vegetation (NC-12A) ash, as well as three fire-impacted soil (NC-1C, NC-6A, and NC-12B) samples (Table S1), were selected to characterize the properties of MNPs of WUI fire ashes from different sources. The ash samples were named as Axxx and NCxxx, referring to the LNU and North Complex fires, with xxx being arbitrary numbers given to the samples. The WUI fire ash samples were collected from two WUI fire sites that burned during the 2020 California fire season, including the North Complex (NC) Fire and the LNU Lightning Complex Fire. Detailed descriptions of these WUI fires and the sample collection are provided elsewhere [7,16,17] and in the Supplementary Information. The ash samples were collected prior to any rain or other precipitation with disposable plastic scoops and placed into reclosable plastic bags. All the fire ashes were homogenized using a mortar and a pestle. The homogenized ashes were sieved using a 10-mesh 2 mm pore size nylon sieve (Zhangxing Instrument, Hangzhou, Zhejiang, China) to remove large particles. The sieved samples were stored in 250 mL acid-washed HDPE bottles in a −20 °C freezer before further treatment.

### 2.2. Magnetic Particles Separation

In an acid-washed 50 mL centrifuge tube, 750 mg of the sieved ash and soil samples were suspended in 30 mL of ultrapure water (UPW). The mixture was mixed via overhead rotation for 2 h at 40 rpm using a multipurpose tube rotator (Fisher Scientific, 88861049, Waltham, MA, USA). The resulting suspensions were bath-sonicated in ice water for 60 min using an ultrasonic bath (Branson 2800, 40 kHz, Danbury, CT, USA) to disperse the particles. The sonicated samples were kept undisturbed for 24 h to remove the large particles via sedimentation. The top 15 mL of supernatant from each ash and soil suspension was pipetted out and transferred into acid-washed 50 mL centrifuge tubes. The transferred suspension was kept undisturbed for 1 h to allow for any remaining large particles to settle. Then, the top 5 mL of supernatant from each ash and soil suspension was pipetted out and transferred into 14 mL polystyrene test tubes (Corning, Falcon 352057, Reynosa, Mexico) for MNPs separation. Another 2 mL of aliquot was pipetted out and transferred into acid-washed 15 mL centrifuge tubes to measure the relaxation rate of the suspension before magnetic particle separation. The relaxation rate of the suspension was measured by a compact Time-Domain Nuclear Magnetic Resonance (TD-NMR) system [31] (Figure S1). The system is made up of custom NMR electronics with a compact and rugged permanent magnet array designed to enable future deployment as an in situ sensor. A detailed description of the TD-NMR system and the method of measuring the relaxation rate using this system is provided elsewhere [31,32].

The magnetic particles were separated using a rudimentary magnetic particle separator, which consisted of an N42 permanent magnet block placed within a 3D-printed frame with slots for inserting test tubes (Figure S2a). Test tubes containing 5 mL of supernatant were placed in the magnetic particle separator for 24 h. The magnetic particle separation was monitored visually by observing the MNPs being attracted to the test tubes’ walls touching the magnet. The water was then decanted carefully from the tubes on the wall opposite the magnet to remove the nonmagnetic particles remaining in the suspension. The MNPs attracted to the tubes’ walls (Figure S2b) were resuspended in 10 mL UPW. The relaxation rate of the ash suspensions after magnetic separation was measured using the TD-NMR system. For each tube, eight scans were collected and averaged to yield one R_2_ measurement. No additional independent replicate tubes were prepared due to the limited sample volume. The instrument-level reproducibility of this compact TD-NMR platform, including a discussion of averaging per measurement, has been reported previously and is cited here for reference [31]. The elemental composition of the magnetically separated MNPs was determined using SP-ICP-TOF-MS. The sample dilutions (1000- to 10,000-fold) in UPW were performed immediately prior to the SP-ICP-TOF-MS analysis and the suspensions were sonicated after dilution to enhance particle dispersion and minimize particle settling during analysis.

### 2.3. Multi-Metal Nanoparticle Composition on Single Particle Basis

The elemental particle composition at the individual particle level was determined by SP-ICP-TOF-MS (TOFWERK, Thun, Switzerland), as described in our previous studies [17,33,34] and summarized in the supplementary information section. The detected NPs were classified into single and multi-metals (smNPs and mmNPs). The mmNPs were further classified into clusters of mmNPs of similar elemental composition using a two-stage (e.g., intra- and inter-sample; Figure S3) automated agglomerative hierarchical clustering analysis performed in MATLAB R2023a, as described elsewhere [33,34,35] and summarized in the supplementary information section. Select elemental ratios were determined on a particle-by-particle basis, taking into account all the particles containing the two elements, and the elemental ratio distribution was determined. The number concentration (NP g^−1^) of the total, smNPs, mmNPs, and cluster members was determined according to SP-ICP-MS theory [36]. The NPs sizes were determined assuming a spherical shape and pure metal oxide phases (Table S3).

### 2.4. Statistical Analysis

Statistical analyses were performed with SAS^®^ version 9.4 software (SAS institute, Cary, NC, USA). Correlation coefficient between log decrease in R_2_ relaxation rate and log nanoparticle number concentration was calculated using Pearson’s correlation method. Fisher’s Z-transformation was used to compute confidence intervals for each correlation, followed by z-tests to calculate *p*-values for correlation comparisons between every two mixture combinations. Particle size distributions of MNPs and elemental ratio distributions within mmNPs in different samples were analyzed using Kolmogorov–Smirnov (K–S) tests with Bonferroni correction for pairwise comparisons of elemental ratio distributions and size distributions among samples within and between soil, structure, and vehicle for each metal. The analysis was implemented using R. In all cases, statistical significance was set at *p*-value < 0.05.

## 3. Results

### 3.1. Efficiency of the Magnetic Separation

**Figure 1:**
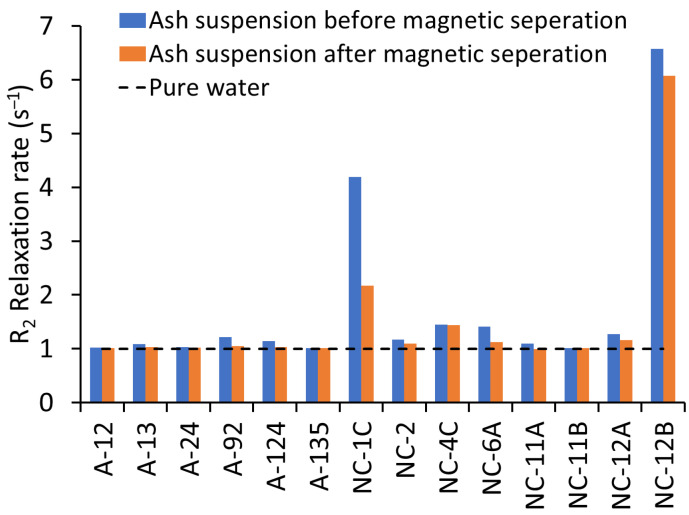
R_2_ relaxation rates of ash suspensions before and after magnetic separation. Higher relaxation rates indicate the presence of magnetic nanoparticles (MNPs) in wildland–urban interface (WUI) fire ashes and fire-impacted soil samples: soil (NC-1C, NC-6A, and NC-12B), vegetation ash (NC-12A), structural ash (A12, A24, A92, A124, A135, and NC-2), and vehicle ash (A13, NC-4C, NC-11A, and NC-11B). The decrease in relaxation rate of ash suspensions after magnetic separation suggests that a portion of the MNPs was successfully isolated.

The R_2_ relaxation rate of the ash suspensions from the WUI fire ashes prior to magnetic separation varied between 1.00 and 6.57 s^−1^ (Figure 1), confirming the presence of MNPs in all the WUI ash samples. An R_2_ relaxation rate close to one indicates low concentrations of MNPs, while values greater than one indicate increased concentrations of MNPs [32]. The R_2_ relaxation rate of the ash suspensions after the magnetic separation of MNPs decreased relative to the corresponding ash suspensions prior to magnetic separation, and approached that of ultrapure water in most of the samples, except for NC-1C, NC-4C, and NC-12B (Figure 1). The decrease in R_2_ relaxation rate of ash suspensions after magnetic separation indicates that some of the MNPs were isolated successfully using the rudimentary magnetic particle separator. The high relaxation rates of NC-1C, NC-4C, and NC-12B ash suspensions after magnetic separation might be attributed to either (1) the sedimentation of particles in these samples during magnetic separation (we observed sediments at the bottom of the test tubes of samples NC-1C and NC-12B; Figure S4); (2) the lack of magnetic separation due to the small size of the NPs or their week magnetism; (3) the relatively weak field of the used magnetic separator; or (4) the high concentration of magnetic particles in these samples, which may have shielded the magnetic field from the magnet. The magnetic separation of weakly magnetic or small-particle NPs can be improved by higher-gradient magnetic setups with agitation or flow-through capture.

The decrease in the relaxation rate following magnetic separation was highest in the fire-impacted soils (0.29–2.02 s^−1^). The decrease in the relaxation rate was much lower in the vehicle (0–0.10 s^−1^), vegetation (0.11 s^−1^), and structural ashes (0–0.17 s^−1^). These differences in the decreases in the relaxation rates might be attributed to the differences in particle or aggregate sizes. Although the same sample mass was used to prepare all the suspensions and all the initial ash and soil suspensions were colored (Table S4), only a few samples—such as NC-1C, A24, NC-6A, NC-12B, and NC-4C—retained visible coloration after the removal of large particles from the suspensions by sedimentation prior to magnetic separation (Figure S5a). After magnetic separation, only the NC-1C and NC-12B suspensions remained visibly colored (Figure S5b), suggesting that a high concentration of suspended particles remained in the non-magnetically separated fractions of these samples. Thus, the high relaxation rates of NC-1C and NC-12B in the non-magnetically separated particle suspensions might be ascribed to the smaller sizes of the MNPs in these samples compared to the other samples.

### 3.2. Particle Number Concentration

**Figure 2:**
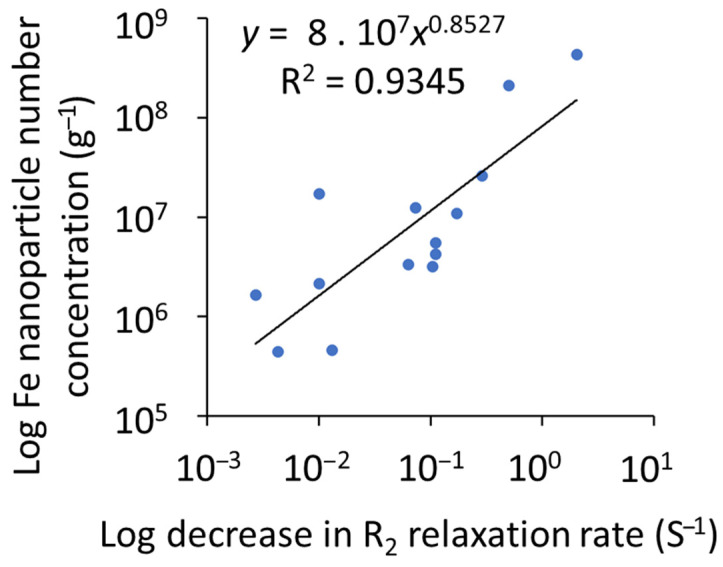
The correlation between the decrease in the R_2_ relaxation rate and the nanoparticles (NPs) number concentration, measured by single particle-inductively coupled plasma–time-of-flight-mass spectrometry (SP-ICP-TOF-MS), in the WUI fire ashes and fire-impacted soil samples. Pearson’s correlation was used to assess the relationship between the log decrease in the R_2_ relaxation rate and the log nanoparticle number concentration (*p* < 0.05).

**Figure 3:**
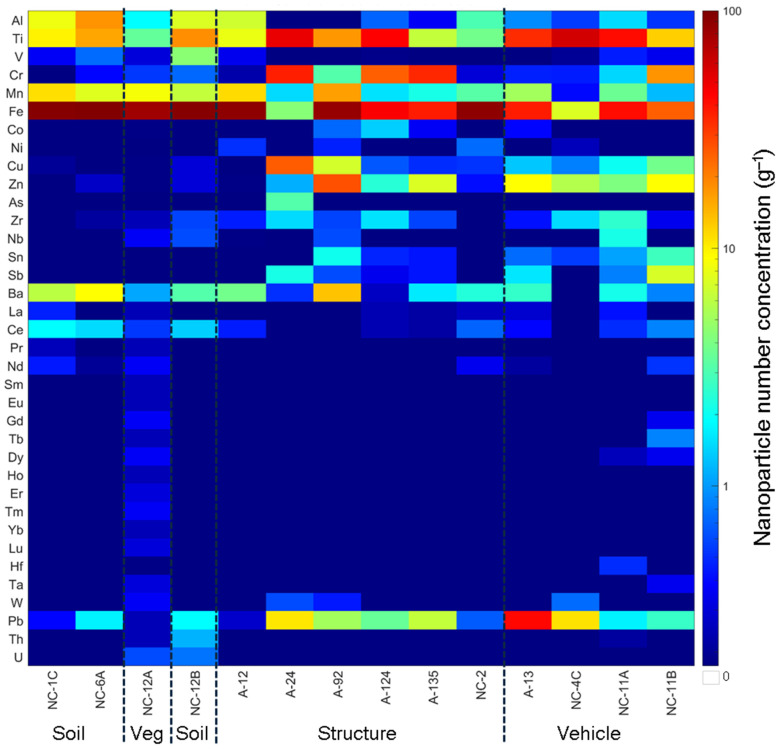
Relative abundance of NPs detected in magnetically separated nanoparticle (MNP) suspensions extracted from WUI fire ash and fire-impacted soil samples: soil (NC-1C, NC-6A, and NC-12B), vegetation (Veg) ash (NC-12A), structural ash (A12, A24, A92, A124, A135, and NC-2), and vehicle ash (A13, NC-4C, NC-11A, and NC-11B).

The total particle number concentration of NPs, including Fe, detected by SP-ICP-TOF-MS in the magnetically separated NP suspensions increased with an increased reduction in the magnetic signal or relaxation rate (R^2^ = 0.9345; Figure 2), suggesting that the Fe-bearing particles were the dominant magnetic particles. The main types of NPs detected in the magnetically separated NPs were Fe-, Ti-, Cr-, Pb-, Mn-, and Zn-bearing NPs (Figure 3). Assuming all the NPs occurred as smNPs, the Fe-bearing NPs accounted for 3.2 to 83.5% of all the magnetically separated NPs and were highest in the vegetation (77.4%) ash and the soil (63.2 to 69.9%). The Ti-bearing NPs accounted for 3.3 to 66.1% of all the magnetically separated NPs and were highest in the vehicle (14.1 to 66.1%) and structural (3.5 to 36.4%) ashes. The Cr-bearing NPs accounted for 0.1 to 34.7% of all the magnetically separated NPs and were highest in the structural (0.2 to 34.7%) and vehicle (0.3 to 20.1%) ashes. The Pb-bearing NPs accounted for 0.2 to 30.5% of all the magnetic NPs and were highest in the vehicle (1.6 to 30.5%) ash. The Cu-bearing NPs accounted for 0 to 18.1% of all the magnetic NPs and were highest in the structural (0 to 18.1%) ash. The Zn-bearing NPs accounted for 0.1 to 14.9% and were highest in the structural (0.1 to 14.9%) and vehicle (3.8 to 10.4%) ashes. The Mn-bearing NPs accounted for 0.5 to 9.3% of all the magnetic NPs and were highest in the vegetation (8.4%) ash and the soil (4.4 to 8.2%). The relative abundance of the different types of NPs in the magnetically separated NPs is consistent with their abundance/use in the fuel sources, as discussed in detail elsewhere [16,37].

### 3.3. Particle Size Distribution

**Figure 4:**
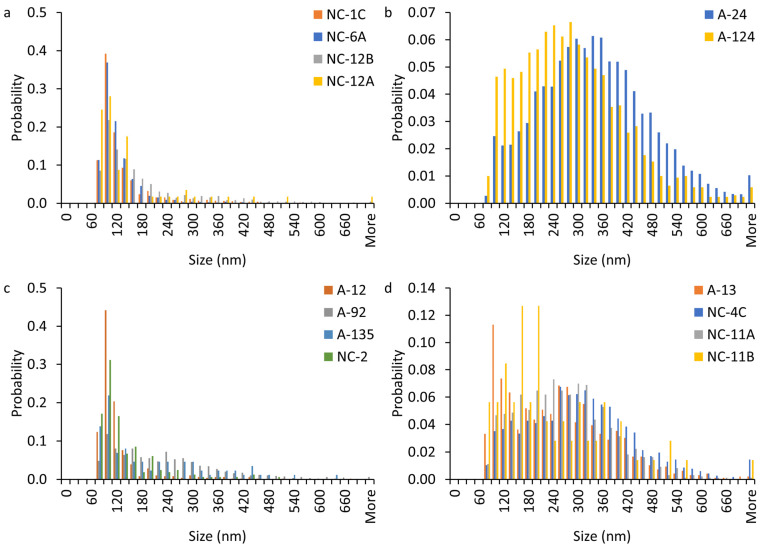
Particle size distribution of Ti-bearing NPs in magnetically separated nanoparticle (MNP) suspensions extracted from WUI fire ash and fire-impacted soil samples assuming pure and spherical TiO_2_ NPs: (**a**) soil (NC-1C, NC-6A, and NC-12B) and vegetation ash (NC-12A), (**b**,**c**) structural ash (A12, A24, A92, A124, A135, and NC-2), and (**d**) vehicle ash (A13, NC-4C, NC-11A, and NC-11B). We apply nonparametric Kolmogorov–Smirnov tests with Bonferroni corrections to investigate if the Ti-bearing particle size distributions are significantly different within and between the soil, structure, and vehicle samples for each metal. There are 73 pairs with significant differences (<0.05) for Ti. Additionally, the size distributions of Ti-bearing particles in 16 out of 24 pairs of structural ash vs. soil and vegetation ashes, 16 out of 16 vehicle ash vs. soil and vegetation ashes, and 21 out of 24 structural ash vs. vehicle ash are significantly different (<0.05).

The Ti-, Cr-, and Zn-bearing NP sizes were smaller in the soil and vegetation ashes than in the structural and vegetation ashes (Figure 4 and Figure S6a–f). These differences are ascribed to the sources of Ti-, Cr-, and Zn-bearing NPs in the fuel material. For instance, the size distribution of Ti-bearing NPs in the soil and vegetation ashes was dominated (56–81%) by particles < 150 nm. In contrast, the size distribution of Ti-bearing NPs in the structural and vehicle ash samples was dominated (86–99%) by larger particles (e.g., 100–700 nm; Figure 4b,d). These larger Ti-bearing NP sizes in the vehicle and structural ashes are consistent with the TiO_2_ pigment particle sizes of paints used in vehicles and structures. These TiO_2_ pigment particle sizes are typically 100–300 nm [38]. The Fe-bearing NP sizes were larger in the vegetation ash and soil than in the structural and vehicle ashes (Figure S6g–i). The size distribution of Fe-bearing NPs in structural and vehicle ashes samples was dominated (63–92%) by particles <160 nm. In contrast, the size distribution of Fe-bearing NPs in soil and vegetation ash samples was dominated (53–85%) by larger particles (e.g., 100–280 nm; Figure S6g).

### 3.4. Particle Composition

#### 3.4.1. smNPs vs. mmNPs

**Figure 5:**
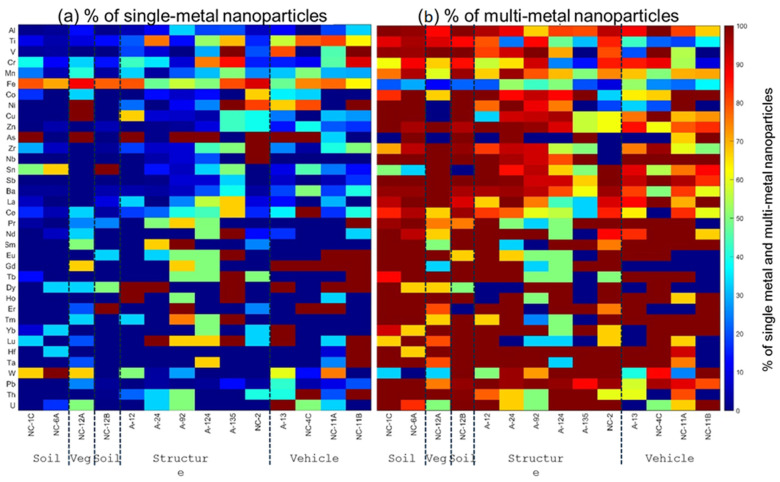
Relative abundance of (**a**) single-metal nanoparticles (smNPs) and (**b**) multi-metal nanoparticles (mmNPs) in magnetically separated nanoparticle (MNP) suspensions extracted from WUI fire ash and fire-impacted soil samples: soil (NC-1C, NC-6A, and NC-12B), vegetation (Veg) ash (NC-12A), structural ash (A12, A24, A92, A124, A135, and NC-2), and vehicle ash (A13, NC-4C, NC-11A, and NC-11B).

The magnetically separated nanoparticles (MNPs) occurred as smNPs or mmNPs (Figure 5). The majority of the detected particles occurred as mmNPs (Figure 5b). A substantial fraction of the detected Al (67 to 100%), Ti (19 to 96%), Mn (50 to 97%), Pb (58 to 100%), Zn (57 to 100%), and Cr (9 to 92%) in the magnetically separated NPs were associated with Fe-bearing NPs, which was ascribed to the aggregation of these NPs with Fe-bearing NPs. However, a notable fraction (3 to 91%) of the detected NPs occurred as smNPs. The smFe-bearing NPs accounted for 48 to 91% of all the Fe-bearing particles. The smTi-bearing NPs accounted for 4 to 81% of all the Ti-bearing particles. The smCr-bearing NPs accounted for 8 to 91% of all the Cr-bearing NPs. The smMn-bearing NPs accounted for 3 to 50% of all the Mn-bearing particles. The smPb-bearing NPs accounted for up to 42% of all the Pb-bearing NPs.

#### 3.4.2. Elemental Ratios in mmNPs

**Figure 6:**
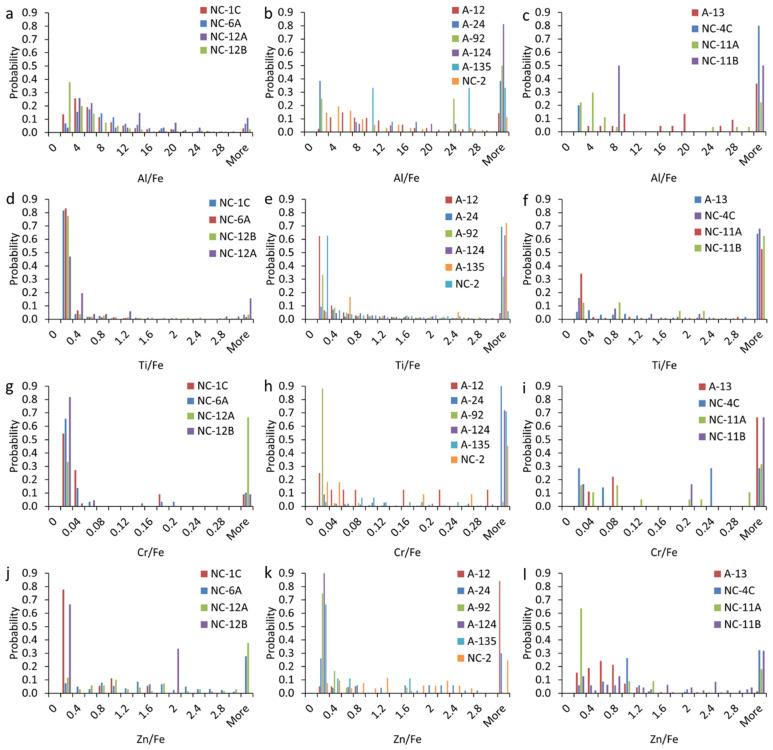
Elemental ratios in multi-metal nanoparticles (mmNPs) in magnetically separated nanoparticle (MNP) suspensions extracted from WUI fire ashes and fire-impacted soil samples: (**a**–**c**) Al/Fe, (**d**–**f**) Ti/Fe, (**g**–**i**) Cr/Fe, and (**j**–**l**) Zn/Fe in (**a**,**d**,**g**,**j**) soil and vegetation ash, (**b**,**e**,**h**,**k**) structural ash, and (**c**,**f**,**i**,**l**) vehicle ash.

The elemental ratios (e.g., Al/Fe, Ti/Fe, Cr/Fe, Zn/Fe, etc.) were higher in the MNPs in the structural and vehicle ashes than those in the soil and vegetation ash; the latter were consistent with the ratios in naturally occurring particles (Figure 6). The elemental ratios of Al/Fe exhibited higher values in the structural and vehicle ashes compared to those in the soil and vegetation ash (Figure 6a–c). The majority (56 to 85%) of Al- and Fe-bearing mmNPs in the soil and vegetation ash were characterized by an Al/Fe ratio between 2 and 10, and were thus typical of those found in natural mmNPs [39]. In contrast, the majority (33 to 94%) of Al- and Fe-bearing mmNPs in the structural and vehicle ashes displayed Al/Fe ratio > 10.

We applied nonparametric Kolmogorov–Smirnov tests with Bonferroni corrections to investigate if the distributions of the elemental ratios were significantly different within and between the soil, structure ash, and vehicle ash for each metal. The results showed that there were 27, 69, 33, and 42 pairs with significant differences (*p* < 0.05) for the Al/Fe, Ti/Fe, Cr/Fe, and Zn/Fe ratios. Additionally, the elemental ratios distributions of Al/Fe in 7 out of 24 pairs of structural ash vs. soil and vegetation ashes, 10 out of 16 vehicle ash vs. soil and vegetation ash pairs, and 7 out of 24 structural ash vs. vehicle ash pairs were significantly different (<0.05). The elemental ratios distributions of Ti/Fe in 21 out of 24 pairs of structural ash vs. soil and vegetation ashes, 16 out of 16 vehicle ash vs. soil and vegetation ash pairs, and 13 out of 24 structural ash vs. vehicle ash pairs were significantly different (<0.05). The elemental ratios distributions of Cr/Fe in 14 out of 24 pairs of structural ash vs. soil and vegetation ash, 3 out of 16 vehicle ash vs. soil and vegetation ashes pairs, and 7 out of 24 structural ash vs. vehicle ash pairs were significantly different (<0.05). The elemental ratios distributions of Zn/Fe in 12 out of 24 pairs of structural ash vs. soil and vegetation ash, 12 out of 16 pairs of vehicle ash vs. soil and vegetation ashes, and 11 out of 24 pairs of structural ash vs. vehicle ash were significantly different (<0.05).

The Ti- and Fe-bearing mmNPs in the soil samples and vegetation ash were characterized by a Ti/Fe ratio of <1.0, and were thus typical of those found in natural NPs (Figure 6d) [33]. In contrast, the structural and vehicle ashes contained a substantial fraction (5 to 78%) of mmNPs with a Ti/Fe ratio >1.0 (Figure 6e,f). The Cr- and Fe-bearing NPs in the soil and vegetation ash were characterized by a Cr/Fe ratio of <0.05. The elemental ratios of Cr/Fe showed higher values in the structural and vehicle ashes compared to the soil samples and vegetation ash (Figure 6g–i). Moreover, the elemental ratios of Zn/Fe displayed higher values in the structural and vehicle ashes compared to the soil samples and vegetation ash (Figure 6j–l).

#### 3.4.3. Multi-Element Nanoparticle Clusters

**Figure 7:**
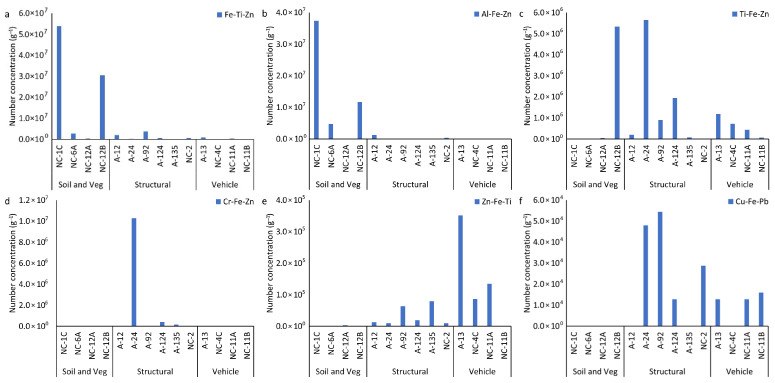
Particle number concentration of the identified multi-metal nanoparticle (mmNP) clusters in magnetically separated nanoparticle (MNP) suspensions extracted from WUI fire ash and fire-impacted soil samples: (**a**) FeTiZn, (**b**) AlFeZn, (**c**) TiFeZn, (**d**) CrFeZn, (**e**) ZnFeTi, and (**f**) CuFePb. The clusters are named using the three most abundant elements (highest-to-lowest mass fraction) within each cluster.

**Figure 8:**
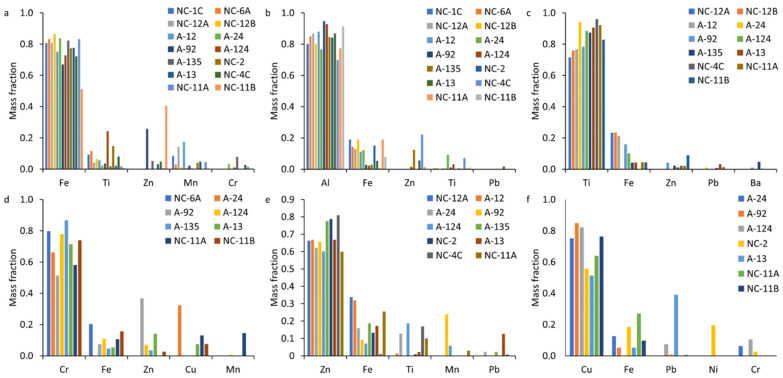
Average elemental composition (mass fraction) of the multi-element nanoparticle (mmNP) clusters identified in the magnetically separated nanoparticle (MNP) suspensions extracted from WUI fire ash and fire-impacted soil samples: (**a**) FeTiZn, (**b**) AlFeZn, (**c**) TiFeZn, (**d**) CrFeZn, (**e**) ZnFeTi, and (**f**) CuFePb. The clusters are named using the three most abundant elements (highest-to-lowest mass fraction) within each cluster: soil (NC-1C, NC-6A, and NC-12B), vegetation ash (NC-12A), vehicle ash (A13, NC-4C, NC-11A, and NC-11B), and structural ash (A12, A24, A92, A124, A135, and NC-2).

Overall, 26 mmNP clusters, named using the three most abundant elements (highest-to-lowest mass fraction) within each cluster, were identified in the soil and ash samples (Figure S7). Many of the clusters were found in very few particles or occurred only in one sample and are not further discussed here. Six major clusters, including FeTiZn, TiFeZn, AlFeCo, CrFeZn, ZnFeTi, and CuFePb, accounted for > 98% of all the mmNPs identified in all the samples (Figure S7). FeTiZn and AlFeZn mmNPs occurred dominantly in the soil and vegetation ash, and accounted for 90% and 96% of mmNPs, respectively (Figure 7a,b). In contrast, TiFeZn, CrFeZn, ZnFeTi, and CuFePb occurred dominantly in the structural and vehicle ashes (Figure 7c–f). In the structural ashes, the dominant clusters were TiFeZn and CuFePb, which accounted for 53% and 78% of mmNPs in all the samples, respectively. The CrFeZn cluster was dominant in structural ash A24, which accounted for 94% of mmNPs (Figure S7). The ZnFeTi cluster was dominant in vehicle ashes, which accounted for 74% of mmNPs. The mean elemental composition of each mmNP cluster is presented in Figure 8. Each mmNP cluster is rich in one type of NP and contains a smaller mass fraction of other NPs. Most of the structural and vehicle ashes contained Ti, Cr, Cu, and Zn mmNP clusters. The elemental composition of mmNP clusters is consistent with the high Al/Fe, Ti/Fe, Cr/Fe, and Zn/Fe ratios found in the structural and vehicle ashes. Iron was detected in all the mmNP clusters. In the Fe-rich mmNP cluster, the mass fraction of Ti was high in A124, NC-2, and NC-4C.

## 4. Discussion

In this study, magnetically separated nanoparticles (MNPs) extracted from fire ashes and fire-impacted soils primarily consisted of Fe-, Ti-, Cr-, Mn-, Zn-, and Pb-bearing NPs. These MNPs occurred both as individual NPs and as aggregated NPs of different types of NPs. The elemental composition of the MNPs varied depending on the source of the fire ash. The Fe-bearing NPs were predominantly found in the soil and vegetation ashes, suggesting a natural source of Fe NPs [33,37], while the Ti-bearing NPs were predominantly present in the structural and vehicle ashes, indicating anthropogenic sources of Ti NPs, such as building materials or coatings [37]. The Pb- and Zn-bearing NPs were also more enriched in the structural and vehicle ashes, consistent with the known use of these metals in buildings and vehicles [37]. The occurrence of different types of NPs, other than the Fe NPs, in the MNPs is attributed to the heteroaggregation of these NPs with magnetic Fe NPs or the sorption of other metals on the Fe NPs during fuel combustion.

The Fe-bearing NP sizes were generally larger in the soil and vegetation ash compared to the structural and vehicle ashes. The differences in the Fe-bearing NP sizes among the various ash types can be attributed to variations in the source materials and combustion conditions. For instance, high-temperature, complete combustion generates smaller particles, whereas lower combustion temperatures, incomplete combustion, and natural weathering processes associated with vegetation and soil promote the aggregation or formation of larger particles [40,41].

The majority of MNPs were in the form of mmNPs, which is ascribed to the occurrence of NPs as multi-metal aggregates in fire ashes or to the sorption of other metals on major elements (e.g., Al, Si, and Fe) during WUI fires. Our previous research demonstrated that NPs in fire ashes occur as aggregates of individual NPs—such as Fe, Mn, Ti, Zn, Cr, Cu, and As—depending on the fuel source, including structures, vehicles, and vegetation [37]. However, certain samples exhibited a notable presence of smNPs, particularly smFe-bearing NPs. The high proportion of smFe may reflect the inherent magnetic properties and abundance of Fe-based NPs in the ashes [7]. Additionally, the high relative abundance of smTi, smMn, smCr, and smPb can be ascribed to disaggregation of NP aggregates after the magnetic separation, as the particles were sonicated again prior to analysis by SP-ICP-TOF-MS.

The elemental ratios of Al/Fe, Ti/Fe, Cr/Fe, and Zn/Fe in the structural and vehicle ashes were higher compared to the soil and vegetation ash. The high elemental ratios of Al/Fe and Cr/Fe in the structural and vehicle ashes indicate the multi-metal aggregation of the pure form of these elements used in vehicle parts and structural materials [37]. Additionally, the high elemental ratios of Ti/Fe and Zn/Fe in the structural and vehicle ashes indicate the anthropogenic contribution of Ti and Zn in the structural and vehicle ashes [37].

Six major clusters, including FeTiZn, TiFeZn, AlFeCo, CrFeZn, ZnFeTi, and CuFePb, accounted for a significant proportion of all the mmNPs identified in the samples (Figure 8). FeTiZn and AlFeZn mmNPs were dominant in the soil samples and vegetation ash, while TiFeZn, CrFeZn, ZnFeTi, and CuFePb mmNPs were predominant in the structural and vehicle ashes. The presence of Fe as a major cluster in the samples increased the efficiency of magnetic separation. For instance, the FeTiZn cluster was dominant in the NC-1C, NC-12B, and NC-6A soil samples. We observed a higher reduction in relaxation rate of the extracted NPs in soil samples NC-1C (2.02 s^−1^), NC-12B (0.50 s^−1^), and NC-6A (0.29 s^−1^), indicating the higher magnetic separation of NPs (Figure 1). In contrast, the presence of elements other than Fe as the major cluster in a sample resulted in (i.e., TiFeZn, CrFeZn, ZnFeTi, and CuFePb) less magnetic separation. For example, the structural ashes (with a relaxation rate decrease of 0–0.17 s^−1^) and vehicle ashes (with a relaxation rate decrease of 0–0.1 s^−1^), which were dominated by TiFeZn, CrFeZn, ZnFeTi, and CuFePb clusters, exhibited a low reduction in relaxation rate of the extracted NPs. These mmNP clusters might have had less Fe in the aggregates, and thus less attraction to the magnet.

Many of the observed NPs exhibited magnetic behavior due to their composition and formation conditions. For instance, Fe, Mn, and Cr oxide phases (i.e., Fe_3_O_4_, γ-Fe_2_O_3_, Mn_3_O_4_, Cr_2_O_3_) are known to be ferrimagnetic or ferromagnetic and are readily attracted to magnets (Table S5) [42,43,44,45,46,47,48,49,50,51,52,53]. During WUI fires, iron oxide particles undergo a reduction process, leading to the formation of magnetic particles, such as maghemite (γ-Fe_2_O_3_), magnetite (Fe_3_O_4_), and zero-valent iron [7]. Jordanova et al. (2019) demonstrated that strong wildfires produce nanometer-sized ferromagnetic or superparamagnetic magnetite and maghemite [22]. Reduced Mn oxides, such as MnO and Mn_3_O_4_, show weak ferromagnetic behaviors at the nanoscale, and thus can be attracted to magnets [49,50]. Cr_2_O_3_ exhibits surface-related weak ferromagnetism when reduced to the nanoscale [51,52], whereas CrO_2_ displays ferromagnetic behavior at room temperature [53].

## 5. Conclusions and Environmental Implications

This study provides novel insights into the composition, size distribution, and elemental associations of MNPs derived from WUI fire ashes. Our findings demonstrate that WUI fire ashes, including structural, vehicle, and vegetation ashes, and fire-impacted soil contain abundant MNPs, primarily composed of Fe, Ti, Cr, Mn, Zn, Pb, and Cu. These MNPs occur both as smNPs and as mmNPs, with Fe-bearing NPs being the most dominant across all the WUI fire ashes. Whereas mmNPs can be attributed to aggregation of NPs during combustion, smNPs could be attributed to their occurrence as single-metal nanoparticles or to disaggregation of NP aggregates during sample preparation by sonication.

A critical and previously underexplored implication of our findings is the association of MNPs with potentially toxic elements (PTEs), such as Cr, Pb, Cu, Mn, and Zn, particularly in mmNPs formed during combustion. This association could exacerbate the environmental and health risks of MNPs, as their magnetic properties may enhance their transport, bioavailability, and persistence in biological systems. For instance, Fe-bearing MNPs (i.e., magnetite) can cross biological barriers; penetrate deep into tissues such as the lungs and brain; and induce oxidative stress, inflammation, and genotoxicity [18,24,54,55,56,57,58,59,60]. The presence of Pb-, Mn-, and Zn-bearing MNPs may further enhance these effects through metal-specific toxicity mechanisms, such as DNA damage, neurotoxicity, or endocrine disruption [15,56,57,58,59,60,61,62]. Moreover, the magnetic behavior of these PTE-associated MNPs may facilitate their retention in tissues with high iron affinity (e.g., brain, liver, and spleen), possibly enabling prolonged cellular exposure and metal accumulation, amplifying their long-term toxicological impact [58,63]. This synergistic effect of magnetism and toxicity presents a compounding risk not typically accounted for in current environmental risk assessments. Therefore, this study highlights the urgent need for environmental risk assessments and toxicological studies on MNPs containing Cr, Pb, Zn, and other PTEs.

From an environmental perspective, MNPs released during WUI fires could alter soil magnetism, disrupt microbial communities, and impact aquatic ecosystems via runoff or atmospheric deposition. For example, Fe- and Mn-bearing MNPs can catalyze redox reactions and promote the formation of reactive oxygen species in aquatic environments [3,28,60,64]. Furthermore, Ti- and Zn-based particles, often used in pigments and coatings, may leach into water systems, affecting algal productivity and nutrient cycling [27,64,65]. By revealing the detailed elemental composition and characteristics of MNPs in WUI fire ashes, this study paves the way for a more nuanced understanding of WUI fire pollution and underscores the importance of addressing MNPs as a distinct class of emerging contaminants with potentially amplified health and environmental consequences.

## Supplementary Materials

The following supporting information can be downloaded at: https://www.mdpi.com/article/10.3390/nano15181420/s1, Table S1. Description of ashes collected following the 2020 fire season; Table S2. Operating conditions for inductively coupled plasma-time of flight-mass spectrometer (ICP-TOF-MS) analysis for single particle analysis modes; Table S3. Elements monitored for single particle-inductively coupled plasma-time of flight-mass spectrometer (SP-ICP-TOF-MS) analysis and the corresponding particle mass and size detection limits; Table S4. Visual description of wildland-urban interface (WUI) fire ash and fire-impacted soil suspensions at different stages: initial suspension, supernatant after sedimentation, non-magnetically separated suspension, and magnetically separated particles suspension; Table S5. Summary of magnetic properties and origins in nanoparticles; Figure S1. Complete setup of the compact TD-NMR system, showing the main components and subsystems with annotations (CC BY-SA 4.0); Figure S2. Magnetic particles (MPs) separation: (a) simple MPs separator which consists of an N42 permanent magnet block placed within a 3D printed frame with slots for inserting test tubes, (b) separated MPs attached on the test tube’s wall; Figure S3. Conceptual diagram of the two-stage hierarchical clustering analysis; Figure S4. Sediments observed at the bottom of the test tubes during the magnetic separation (a) NC-1C and (b) NC-12B; Figure S5. Visual color of the suspended particle suspensions (a) after removing the large particles from the suspensions and before magnetic separation and (b) after magnetic separation; Figure S6. Particle size distribution of (a–c) Cr-bearing nanoparticles (NPs), (d–f) Zn-bearing NPs, and (g–i) Fe-bearing NPs in magnetically separated nanoparticle (MNP) suspensions extracted from WUI fire ash and fire-impacted soil samples: (a,d,g) soil and vegetation ash, (b,e,h) structural ash, and (c,f,i) vehicle ash. We applied the nonparametric Kolmogorov–Smirnov tests with Bonferroni correction to investigate if the Ti-bearing particle size distributions were significantly different within and between soil, structure and vehicle for each metal. There are 21 pairs with significant differences (<0.05) for Cr. Additionally, the size distributions of Cr-bearing particles in 7 out of 24 pairs of structural ash vs. soil and vegetation ash, 1 out of 16 vehicle ash vs. soil and vegetation ash, and 5 out of 24 structural ash vs. vehicle ash were significantly different (<0.05). There are 21 pairs with significant differences (<0.05) for Zn. Additionally, the size distributions of Zn-bearing particles in 4 out of 24 pairs of structural ash vs. soil and vegetation ash, 4 out of 16 vehicle ash vs. soil and vegetation ash, and 7 out of 24 structural ash vs. vehicle ash were significantly different (<0.05). There are 77 pairs with significant differences (<0.05) for Fe. Additionally, the size distributions of Fe-bearing particles in 22 out of 24 pairs of structural ash vs. soil and vegetation ash, 13 out of 16 vehicle ash vs. soil and vegetation ash, and 19 out of 24 structural ash vs. vehicle ash were significantly different (<0.05); Figure S7. Number concentration of NPs identified across all elemental clusters in magnetically separated nanoparticle (MNP) suspensions extracted from WUI fire ash and fire-impacted soil samples: soil (NC-1C, NC-6A, NC-12B), vegetation (Veg) ash (NC-12A), structural ash (A12, A24, A92, A124, A135, NC-2), and vehicle ash (A13, NC-4C, NC-11A, NC-11B). The color scale represents the particle number concentration (mL-1) on a logarithmic scale. References [66,67,68,69] are cited in the supplementary materials.
