# Supplementary material for: Elemental Composition of Magnetic Nanoparticles in Wildland–Urban Interface Fire Ashes Revealed by Single Particle-Inductively Coupled Plasma-Time-of-Flight-Mass Spectrometer
Source: Nanomaterials (Basel). 2025 Sep 15;15(18):1420. doi: 10.3390/nano15181420 (PMC12472571; doi:10.3390/nano15181420)
Supplement: Supplementary file 1 [file nanomaterials-15-01420-s001.zip › nanomaterials-3819936-supplementary.pdf]

Supporting Information for

Elemental Composition of Magnetic Nanoparticles in Wildland–Urban Interface Fire Ashes Revealed by Single Particle-Inductively Coupled Plasma-Time-of-Flight-Mass Spectrometer

Mahbub Alam [1], Austin R. J. Downey [2,3], Bo Cai [4] and Mohammed Baalousha [1,*]

[1] Center for Environmental Nanoscience and Risk, Department of Environmental Health Sciences, Arnold School of Public Health, University of South Carolina, Columbia, SC 29208, USA; mahbub@email.sc.edu
[2] Department of Mechanical Engineering, University of South Carolina, Columbia, SC 29208, USA; austindowney@sc.edu
[3] Department of Civil and Environmental Engineering, University of South Carolina, Columbia, SC 29208, USA
[4] Department of Epidemiology and Biostatistics, Arnold School of Public Health, University of South Carolina, Columbia, SC 29208, USA; bocai@mailbox.sc.edu
* Correspondence: mbaalous@mailbox.sc.edu

1. Description of the sampled WUI fires

The North Complex (NC) Fire was the seventh largest in California history and the second largest recorded in the northern Sierra Nevada. The NC fire burned 1,290 km^2 and destroyed 2,455 structures in the Feather River watershed northeast of Lake Oroville, California, between August 17 and December 3, 2020. The West Zone of the NC fire burned over 342 km^2 located primarily within the Plumas National Forest. The distribution of burn severity within this fire was 2% low, 8% moderate, and 89% high [66]. Fire ash and soil samples were collected in the Berry Creek community, where most of the structures were destroyed. The LNU Lightning Complex Fire was the sixth-largest in California history. It burned 1,470 km^2 and destroyed 1,491 structures in Colusa, Lake, Napa, Sonoma, Solano, and Yolo Counties, approximately 60 km west of Sacramento, between August 17 and October 2, 2020 [67]. Within the fire perimeter, the distribution of burn severity was 12% low, 39% moderate, and 49% high. Land use in the fire perimeter comprised of 57% shrub/scrub, 19% herbaceous, 12% evergreen forest, and 1.4% developed.

Table S1. Description of ashes collected following the 2020 fire season.

Sample Number	Sampling Date	Ash Source	Ash Color	Sample Description
**Lightening Complex Fire samples**				
A12	10/7/2020	Structure/Residential	White	Burned shed - replicate
A13	10/7/2020	Vehicle	Black	Burned trailer - south end
A24	10/7/2020	Structure/Foundation	Green	Dark green material on foundation (Cu arsenate?)
A92	10/15/2020	Structure/Residential	Red/Brown	Red soil - artificial wood from deck
A124	10/15/2020	Structure/Residential/paint	Gray/Brown	Shed - house paint, some wires
A135	10/16/2020	Structure/vehicle	Gray	Burned farm workshop - tools, Cu wire, tractor, tires; some blue pigments 2 shades), black, white, red & brown ash; white powder
**North Complex Fire samples**				
NC-1C	10/7/2020	Soil	Red/Brown	Scraped ash away from surface soil and collect 0-2 cm. Soil was a clay rich, red-orange color, very dense. We could not dig down with the plastic scoop (dry clay)
NC-2	10/7/2020	Structure/Residential	Gray/Brown	House "ash" collected from bedroom or living room area. Removed large tile material and collected from below large pieces
NC-4C	10/7/2020	Vehicle	Black	Tire material (collected in glass for organic analysis)
NC-6A	10/7/2020	Soil	Red/Brown	Soil 0-2 cm
NC-11A	10/7/2020	Vehicle	Gray	Car ash, composite from ground, early 2000's Ford Mustang
NC-11B	10/7/2020	Vehicle	Gray	Car ash, interior
NC-12A	10/7/2020	Vegetation	Black	Ash from pine forest. High severity. Above reservoir.
NC-12B	10/7/2020	Soil	Red/Brown	Soil 0-2 cm

Ash and soil samples (NC-12) were collected from the location representing slightly different soil composition. The NC-12 sample was collected from a severely burned east-facing slope approximately 1.5 km north of Madrone Lake and in the north arm tributary to Madrone Lake. The ash samples (NC-12A) were first scraped and collected from the surface.

Table S2. Operating conditions for inductively coupled plasma-time of flight-mass spectrometer (ICP-TOF-MS) analysis for single particle analysis modes.

Instrument Parameter	Value				
Plasma Power	1550 V				
Nebulizer Gas Flow	1.1-1.14 L/min				
Auxiliary Gas Flow	0.8 L/min				
Cooling Gas Flow	14 L/min				
Injector Diameter	2.5 mm				
Collision Cell Gas	5 mL/min He with 4.5% H_2				
CCT Bias	-2.50 V				
Notch	Mass	29	32	36.3	41
	Amplitude (V)	1.6	2.0	2.0	1.2

Data Acquisition	Continuous Mode
Detected Mass Range	14-275 m/Z
TOF Repetition Rate	33 kHz
TOF Time Resolution	30 µs
Integration Time	2 ms
Acquisition Time	200-300 s
Sample Flow Rate	0.455 mL/min
Transport Efficiency	6.6% (5-7%)
(CeO/Ce)	< 3.0%

2. *Nanoparticle Composition on Single Particle Basis*

The elemental particle composition at the individual particle level was determined by SP-ICP-TOF-MS (TOFWERK, Thun, Switzerland) as described in our previous studies [17,33,34]. Samples were introduced into the ICP with a 2DX autosampler (Element Scientific, Omaha, United States) and a MicroMist U-series Nebulizer (Thermo Scientific, USA) connected via a Quartz Cyclonic Spray Chamber (Meinhard, USA) to the injector of the ICP torch. The instrument operating parameters and the monitored isotopes are summarized in **Tables S2 and S3**, respectively. Element-specific instrument sensitivities were measured with a series of multi-element solutions prepared from a mixed multi-element ICP certified reference standard (0, 1, 2, 5, and 10 μgL^{-1} multi-element standard, diluted in 1% HNO_3, BDH Chemicals, Radnor, PA, USA). The transport efficiency was calculated via the known size method using a certified 60-nm Au ENMs (NIST RM 8013 Au, Gaithersburg, MD, USA) and a series of ionic Au standards (BDH Chemicals, West Chester, PA, USA) [36]. A 4.5% H_2/He gas mixture was used as collision gas to eliminate/minimize interferences and was optimized for $^{56}Fe^{+}$ and $^{28}Si^{+}$ signals. All data processing – signal thresholding (Poisson algorithm [68]) and split event correction - was performed using Tofpilot (Version 2.11.3, TOFWERK, Thun, Switzerland). The mass and size detection limits, assuming pure metal and metal oxide phases, are summarized in **Table S3**. All samples and UPW blanks were analyzed in triplicate and data was acquired for 200 s for each replicate. After verifying the reproducibility of the single particle elemental composition and number concentrations among the replicates, the three replicates were combined to achieve a comprehensive analysis due to limited detection events of certain elements.

3. *Clustering Analysis of Multi-Metal Nanoparticles*

The detected NPs were classified into single- and multi-metals (smNPs and mmNPs). The mmNPs were further classified into clusters of mmNPs of similar elemental composition using a two-stage (e.g., intra- and inter-sample, **Figure S3**) automated agglomerative hierarchical clustering analysis performed in MATLAB as described elsewhere [33-35]. The first stage (intra-sample) clustering allows grouping mmNPs with similar elemental compositions within each sample. The second stage (inter0sample) clustering allows comparing the clusters of mmNPs identified in the first stage across samples. Intra-sample clustering was performed on all metal masses in each NP, using average correlation distance, to generate clusters that best account for variance in NP metallic composition in each sample. The generated clusters were grouped into major clusters using an optimal distance cutoff. A cluster representative was determined for each major cluster as the mean of metal mass in individual NPs within each cluster, taking into account all elements that occurred in at least 5% of NPs within the cluster. The mean intra-sample cluster composition was determined as the mean of the metal mass fraction in all NPs in the cluster and was

compared across samples. Inter-sample clustering was performed on the major cluster representatives identified in the intra-sample clustering to group/cluster the similar NP major clusters identified in the different samples. Major/similar clusters were identified using an optimal cutoff.

The optimal distance cutoff was determined by maximizing the mean silhouette score for each sample. The silhouette score is a measure of how similar an NP is to its own cluster (cohesion) compared to other clusters (separation) [69]. Thus, the silhouette coefficient provides a measure of how well each NP has been classified [69]. The silhouette score ranges from −1 to +1, where a high value indicates that an NP is well matched to its own cluster and poorly matched to neighboring clusters (far away from the neighboring clusters). In contrast, 0 indicates that an NP is on or very close to the decision boundary between two neighboring clusters. A negative value indicates that an NP might have been assigned to the wrong cluster. For the first stage of hierarchical clustering, major clusters were determined for a range of distance cutoffs from 0.3 to 0.9 with an increment of 0.05. Then, the mean silhouette coefficient was determined for each distance cutoff. The optimal distance cutoff was selected as that resulting in the highest mean silhouette score. For the second stage hierarchical clustering, the distance cutoff values varied between 0.02 and 0.6 with an increment of 0.001.

Select elemental ratios were determined on a particle-by-particle basis, taking into account all particles containing the two elements, and the elemental ratio distribution was determined. The number concentration (NP g^{-1}) of the total, smNPs, mmNPs, and cluster members was determined according to SP-ICP-MS theory [36]. The NP particle sizes were determined assuming spherical shape and pure metal oxide phases (**Table S3**).

Table S3. Elements monitored for single particle-inductively coupled plasma-time of flight-mass spectrometer (SP-ICP-TOF-MS) analysis and the corresponding particle mass and size detection limits.

Element	Isotope	Mass Detection Limit (g)	Size Detection Limit (nm, Assuming Pure Metallic Particle)	Size Detection Limit (nm, Assuming Pure Metal Oxide Particle)	Metal Oxide Form
Al	^{27}Al	6.5-6.8 × 10^{-14}	357-363	389-396	Al_2O_3
Ti	^{48}Ti	7.6-8.3 × 10^{-16}	69-71	83-85	TiO_2
V	^{51}V	4.7 × 10^{-16}	53	78	V_2O_5
Cr	^{52}Cr	4.5-4.8 × 10^{-16}	49-50	62-63	Cr_2O_3
Mn	^{55}Mn	3.0-3.1 × 10^{-16}	43-44	56-57	MnO_2
Fe	^{56}Fe	4.8-5.3 × 10^{-16}	49-50	63-65	Fe_2O_3
Co	^{59}Co	2.8 × 10^{-16}	39	49	Co_3O_4

Ni	^{60}Ni	1.2×10^{-15}	64	77	NiO
Cu	^{65}Cu	1.0×10^{-15}	60-61	73	CuO
Zn	^{66}Zn	1.9×10^{-15}	79-80	93-94	ZnO
Zr	^{90}Zr	3.1×10^{-16}	45	52	ZrO_2
Nb	^{93}Nb	1.5×10^{-16}	32	44	Nb_2O_5
Sn	^{120}Sn	3.0×10^{-16}	43	47	SnO_2
Sb	^{121}Sb	3.9×10^{-16}	48	55	Sb_2O_3
Ba	^{138}Ba	$1.2\text{-}1.3 \times 10^{-16}$	41	36	BaO
La	^{139}La	$8.4\text{-}8.7 \times 10^{-17}$	30	31	La_2O_3
Ce	^{140}Ce	$9.3\text{-}9.5 \times 10^{-17}$	30-31	31-32	CeO_2
Pr	^{141}Pr	7.4×10^{-17}	28	30	Pr_6O_{11}
Nd	^{142}Nd	2.0×10^{-16}	38	40	Nd_2O_3
Gd	^{158}Gd	$2.7\text{-}2.9 \times 10^{-16}$	40-41	43-44	Gd_2O_3
Tb	^{159}Tb	$6.0\text{-}6.1 \times 10^{-17}$	24	26-27	Tb_4O_7
Dy	^{164}Dy	2×10^{-16}	36	39	Dy_2O_3
Ho	^{165}Ho	$5.9\text{-}6.1 \times 10^{-17}$	23-24	25	Ho_2O_3
Er	^{166}Er	$1.8\text{-}1.9 \times 10^{-16}$	34	36	Er_2O_3
Tm	^{169}Tm	6.0×10^{-17}	23	25	Tm_2O_3
Lu	^{175}Lu	$5.2\text{-}5.4 \times 10^{-17}$	22	23	Lu_2O_3
Hf	^{180}Hf	1.6×10^{-16}	29	34	HfO_2
Ta	^{181}Ta	6.6×10^{-16}	20	27	Ta_2O_5
W	^{184}W	2.4×10^{-16}	29	37	WO_2
Pb	^{208}Pb	$1.3\text{-}1.4 \times 10^{-16}$	28-29	31	PbO
Th	^{232}Th	$6.4\text{-}6.5 \times 10^{-17}$	22	24	ThO_2
U	^{238}U	$6.3\text{-}6.6 \times 10^{-17}$	18-19	23	UO_2

Particle mass detection limit is calculated according to the Poisson distribution = $Mass_{detection\ limit} = 3.29\sqrt{background\ signal} + 2.71$

Size detection limit is calculated as the equivalent spherical diameter from the particle mass detection limit assuming pure metal and metal oxide phases.

Table S4. Visual description of wildland-urban interface (WUI) fire ash and fire-impacted soil suspensions at different stages: initial suspension, supernatant after sedimentation, non-magnetically separated suspension, and magnetically separated particles suspension.

Samples	Color of the Initial Suspension	Color of the Supernatant After Sedimentation	Color of the Non-Magnetically Separated Suspension	Color of the Magnetically Separated Particles Suspension
A12	White	Transparent	Transparent	Transparent
A13	Black	Turbid/ Transparent	Turbid/ Transparent	Transparent
A24	Green	Green	Green	Green sediment observed after several days
A92	Red/Brown	Red/Brown	Reddish/Turbid	Reddish sediment observed after several days
A124	Red/Gray	Red/Gray	Gray/ Transparent	Transparent
A135	Gray	Turbid/ Transparent	Turbid/ Transparent	Transparent
NC1-C	Red/Brown	Red/Brown	Red/Brown	Red/Brown
NC2	Red/Gray	Turbid/ Transparent	Turbid/ Transparent	Transparent
NC4-C	Black	Black	Black	Transparent
NC6-A	Red/Brown	Brown	Brownish	Reddish/Brownish sediment observed after several days
NC11-A	Gray	Turbid/ Transparent	Turbid/ Transparent	Transparent
NC11-B	Gray	Turbid	Turbid/ Transparent	Transparent
NC12-A	Black	Turbid/ Transparent	Turbid/ Transparent	Transparent
NC12-B	Red/Brown	Red/Brown	Red/Brown	Red/Brown

Table S5. Summary of magnetic properties and origins in nanoparticles.

Nanoparticles	Typical Magnetic Behavior	Origin of Magnetism	Remarks	References
**Magnetite (Fe_3O_4)**	Strongly ferrimagnetic or ferromagnetic (size-dependent)	Fe^{2+}–Fe^{3+} exchange interaction	Strong magnetism at room temperature; size-sensitive	[42-44]
**Maghemite (γ-Fe_2O_3)**	Ferrimagnetic or ferromagnetic	Fe^{3+}–Fe^{3+} superexchange	Strong magnetism at room temperature; common in oxidized nanoparticles	[44-46]
**Metallic Fe/Ni/Co**	Ferromagnetic (bulk and nanoscale)	Spin exchange interaction among metallic atoms	High saturation magnetization	[47,48]
**Reduced Mn oxide (MnO, Mn_3O_4)**	Antiferromagnetic or weak ferromagnetic	Mn^{2+}–Mn^{3+} exchange, surface effects	Magnetic properties at nanoscale	[49,50]
**Chromium dioxide (CrO_2, Cr_2O_3)**	Antiferromagnetic (bulk); weak ferromagnetic in nanoparticles	Cr^{3+}–Cr^{3+} antiferromagnetic exchange; surface effects	Surface effects cause weak ferromagnetic in nanoscale, ferromagnetic behavior at room temperature	[51-53]

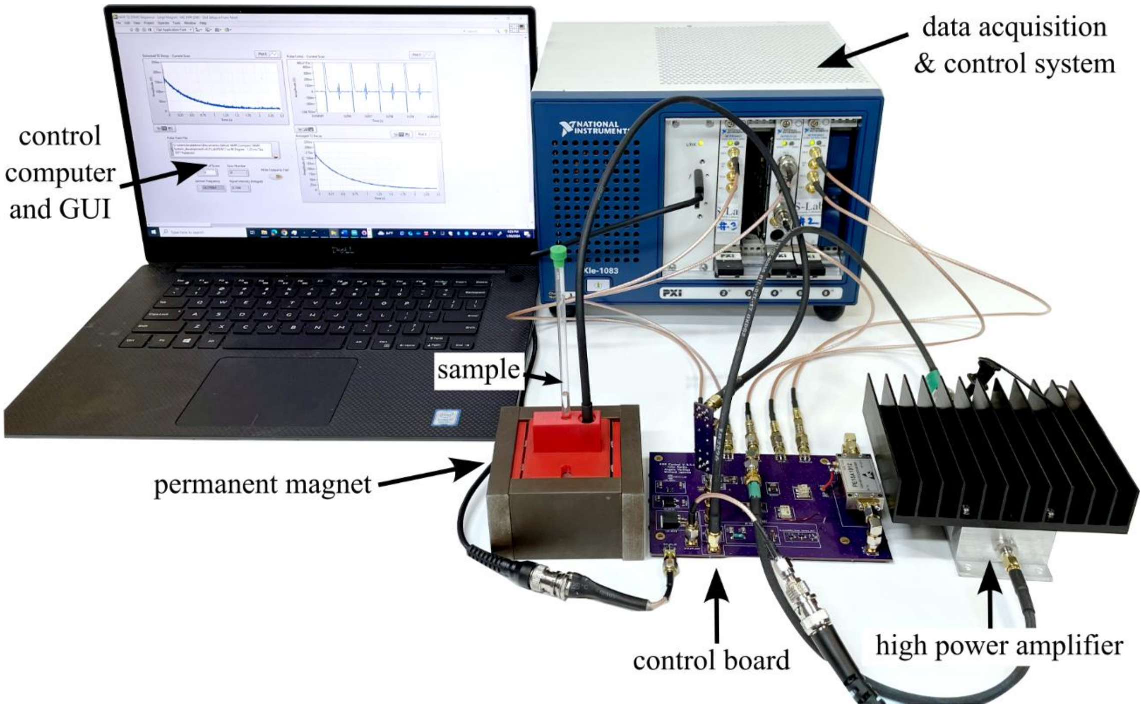

Figure S1. Complete setup of the compact TD-NMR system, showing the main components and subsystems with annotations (CC BY-SA 4.0) [31].

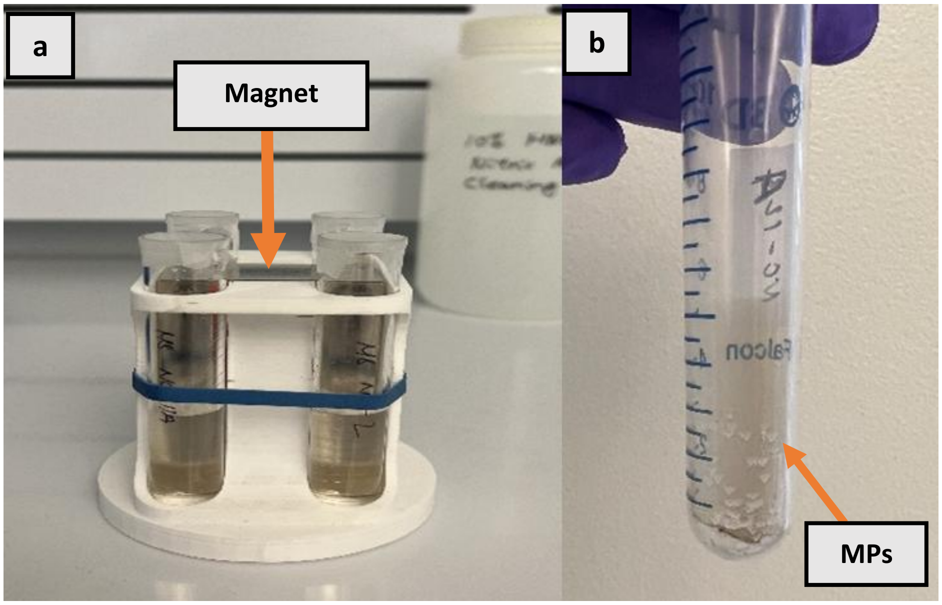

Figure S2. Magnetic particles (MPs) separation: (a) simple MPs separator which consists of an N42 permanent magnet block placed within a 3D printed frame with slots for inserting test tubes, (b) separated MPs attached on the test tube's wall.

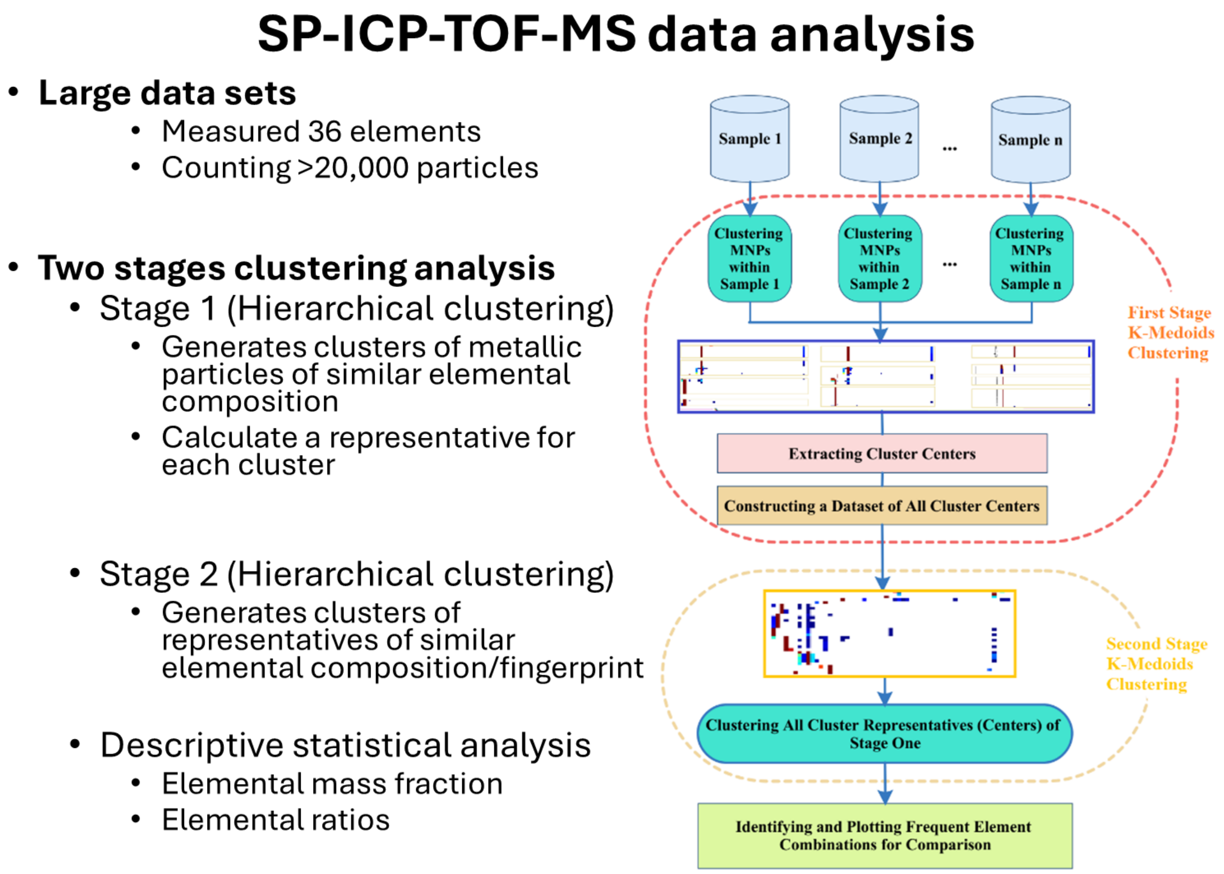

Figure S3. Conceptual diagram of the two-stage hierarchical clustering analysis.

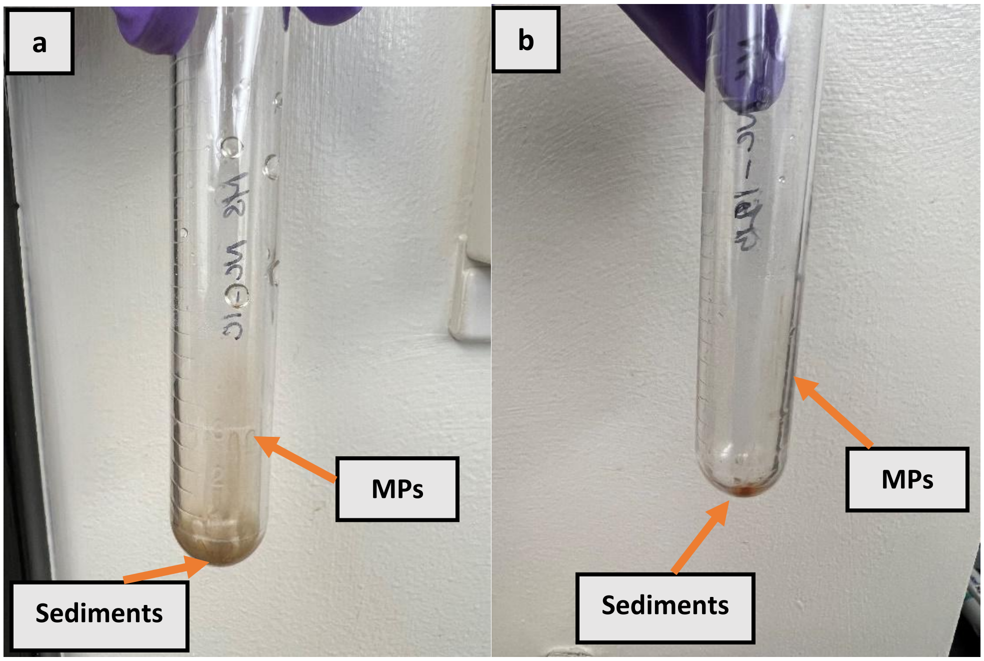

Figure S4. Sediments observed at the bottom of the test tubes during the magnetic separation (a) NC-1C and (b) NC-12B.

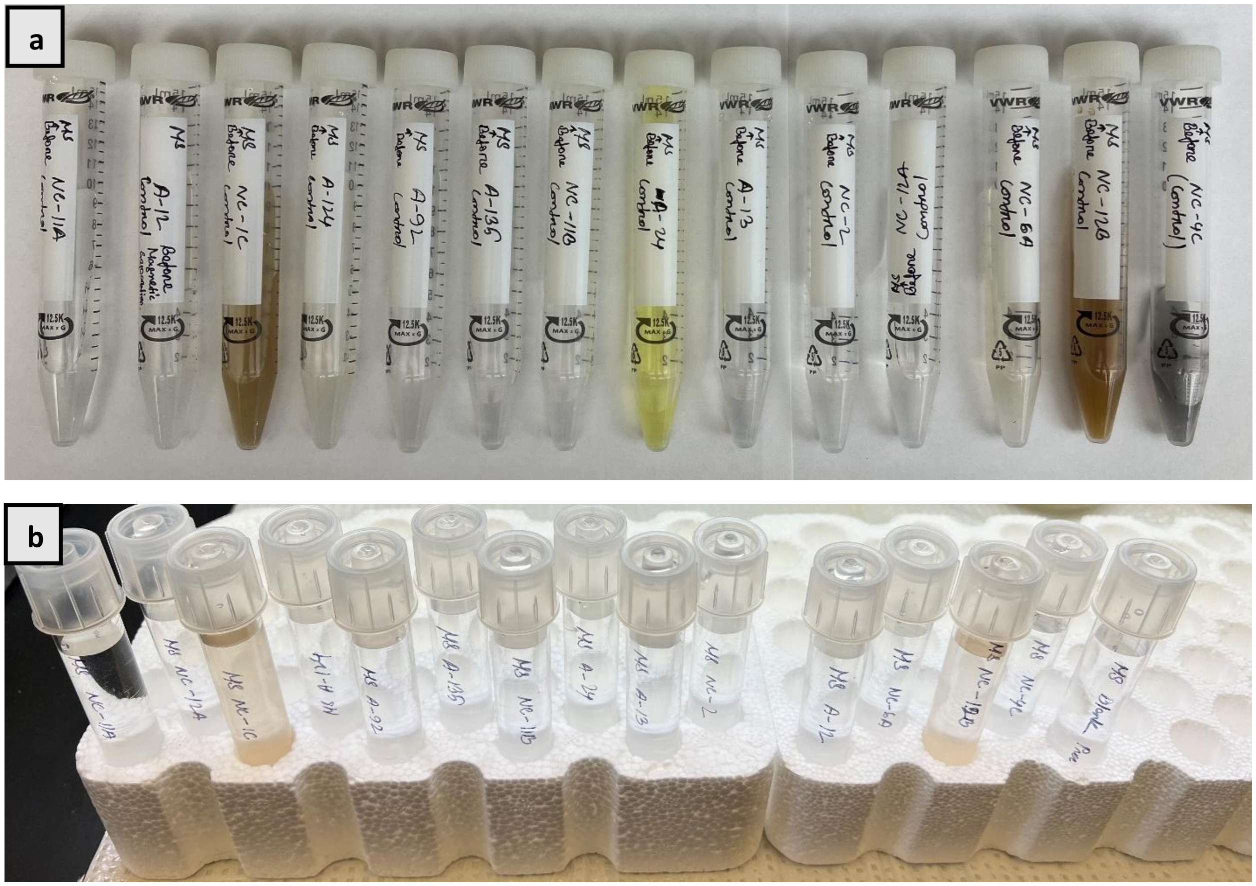

Figure S5. Visual color of the suspended particle suspensions (a) after removing the large particles from the suspensions and before magnetic separation and (b) after magnetic separation.

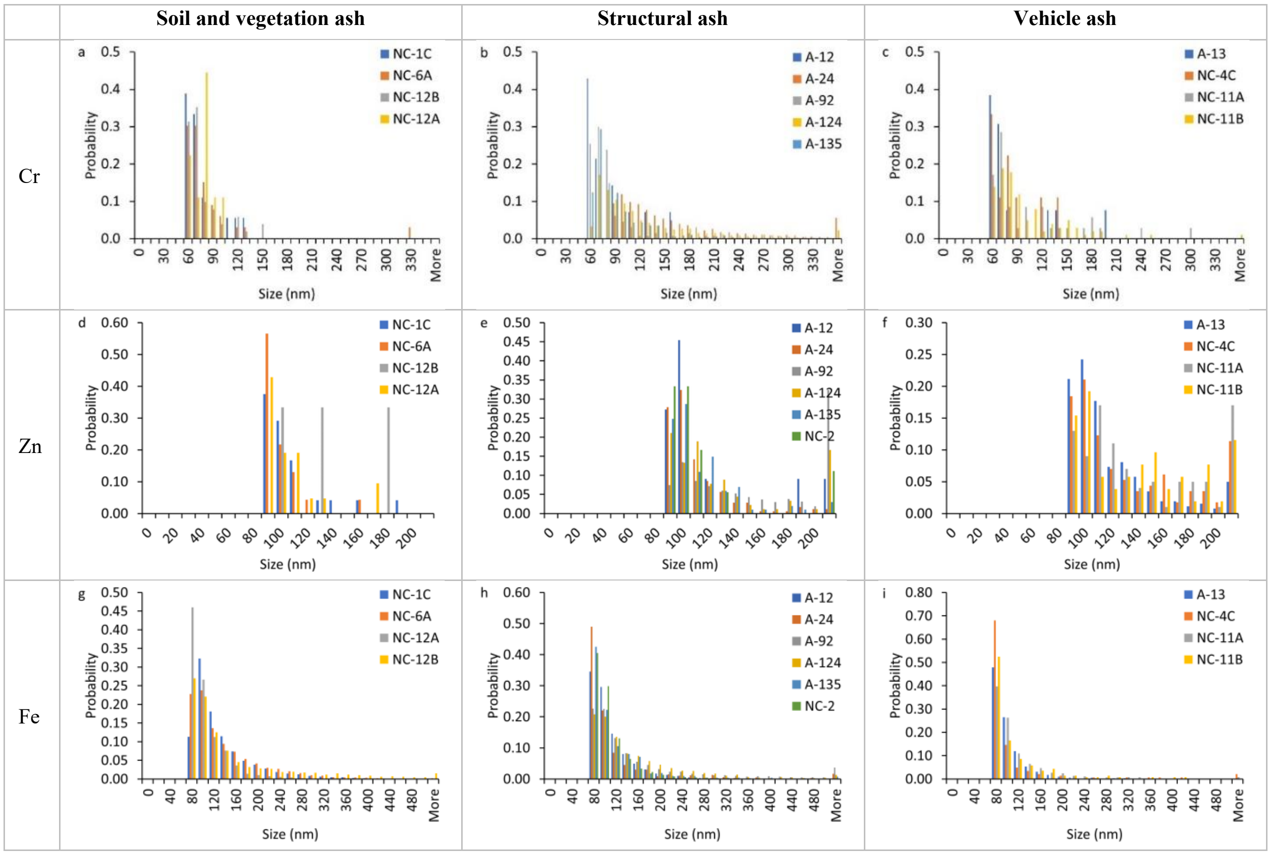

Figure S6. Particle size distribution of (a-c) Cr-bearing nanoparticles (NPs), (d-f) Zn-bearing NPs, and (g-i) Fe-bearing NPs in magnetically separated nanoparticle (MNP) suspensions extracted from WUI fire ash and fire-impacted soil samples: (a, d, g) soil and vegetation ash, (b, e, h) structural ash, and (c, f, i) vehicle ash. We applied the nonparametric Kolmogorov–Smirnov tests with Bonferroni correction to investigate if the Ti-bearing particle size distributions were significantly different within and between soil, structure and vehicle for each metal. There are 21 pairs with significant differences (< 0.05) for Cr. Additionally, the size distributions of Cr-bearing particles in 7 out of 24 pairs of structural ash *vs.* soil and vegetation ash, 1 out of 16 vehicle ash *vs.* soil and vegetation ash, and 5 out of 24 structural ash *vs.* vehicle ash were significantly different (< 0.05). There are 21 pairs with significant differences (< 0.05) for Zn. Additionally, the size distributions of Zn-bearing particles in 4 out of 24 pairs of structural ash *vs.* soil and vegetation ash, 4 out of 16 vehicle ash *vs.* soil and vegetation ash, and 7 out of 24 structural ash *vs.* vehicle ash were significantly different (< 0.05). There are 77 pairs with significant differences (< 0.05) for Fe. Additionally, the size distributions of Fe-bearing particles in 22 out of 24 pairs of structural ash *vs.* soil and vegetation ash, 13 out of 16 vehicle ash *vs.* soil and vegetation ash, and 19 out of 24 structural ash *vs.* vehicle ash were significantly different (< 0.05).

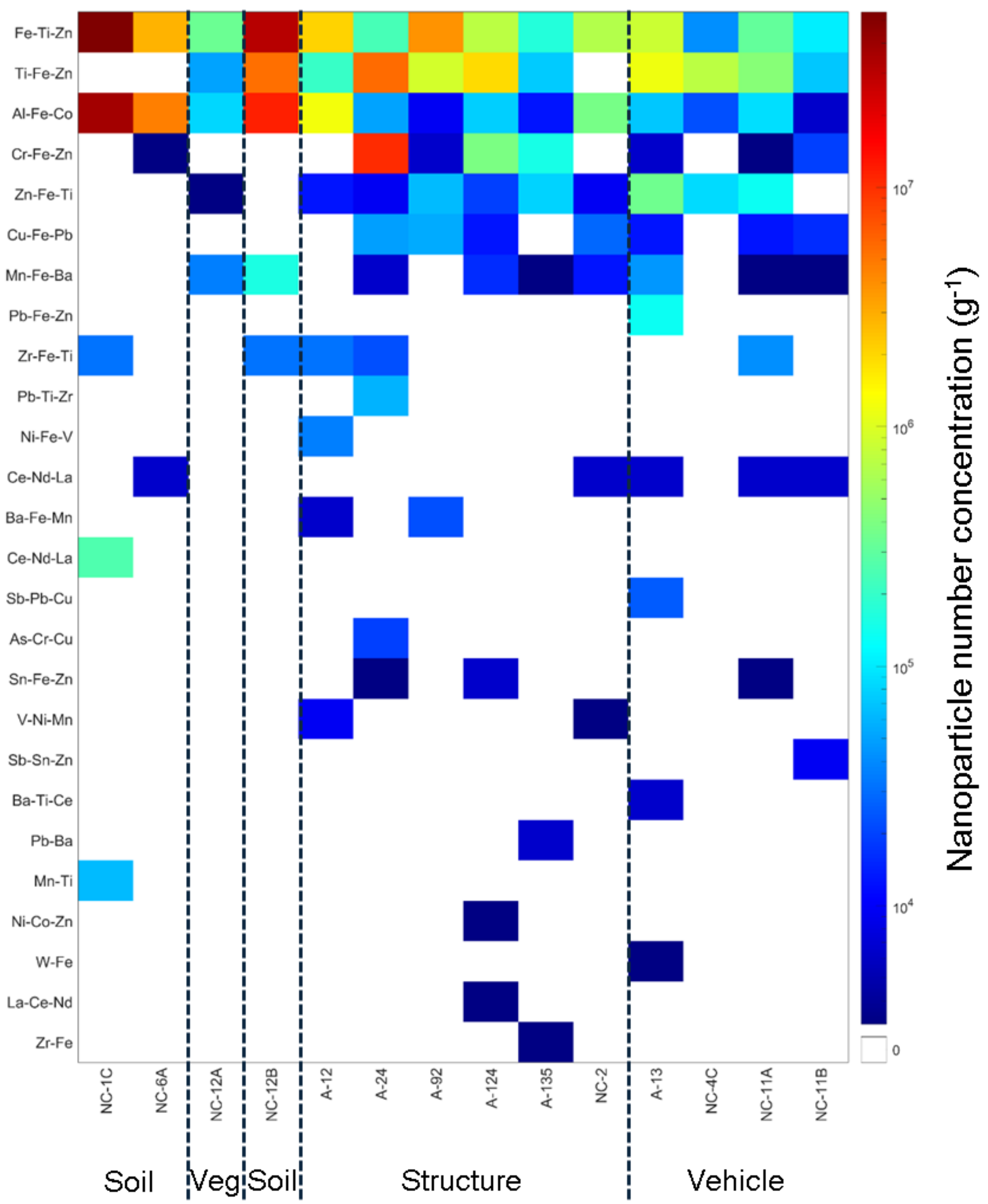

Figure S7. Number concentration of NPs identified across all elemental clusters in magnetically separated nanoparticle (MNP) suspensions extracted from WUI fire ash and fire-impacted soil samples: soil (NC-1C, NC-6A, NC-12B), vegetation (Veg) ash (NC-12A), structural ash (A12, A24, A92, A124, A135, NC-2), and vehicle ash (A13, NC-4C, NC-11A, NC-11B). The color scale represents the particle number concentration (mL^{-1}) on a logarithmic scale.
